# ﻿An integrative approach to a revision of the freshwater mussel genus *Songkhlanaia* (Bivalvia, Unionidae), with the description of a new species

**DOI:** 10.3897/zookeys.1224.140549

**Published:** 2025-01-28

**Authors:** Ekgachai Jeratthitikul, Chirasak Sutcharit, Pongpun Prasankok

**Affiliations:** 1 Animal Systematics and Molecular Ecology Laboratory, Department of Biology, Faculty of Science, Mahidol University, Bangkok 10400, Thailand Mahidol University Bangkok Thailand; 2 Animal Systematics Research Unit, Department of Biology, Faculty of Science, Chulalongkorn University, Bangkok 10330, Thailand Chulalongkorn University Bangkok Thailand; 3 School of Biology, Institute of Science, Suranaree University of Technology, Nakhon Ratchasima 30000, Thailand Suranaree University of Technology Nakhon Ratchasima Thailand

**Keywords:** Freshwater mussels, Indochina, Mekong Basin, multi-locus phylogeny, new taxa, Pseudodontini, taxonomic revision, Thailand

## Abstract

Mainland Southeast Asia, also known as Indochina, is recognized as a global biodiversity hotspot for freshwater mussels, hosting a significant number of species and exhibiting high levels of endemism. Recently, the monotypic genus *Songkhlanaia* was described from the Songkhla Lake Basin in southern Thailand. In this study, two additional lineages are revealed, *S.moreleti***comb. nov.** and *S.songkhramensis***sp. nov.**, from the Mekong Basin through an integrative taxonomic approach combining morphological characters and molecular phylogenetic analyses. The multi-locus phylogenetic inference supported the monophyly of the genus and further elucidated the sister relationship between *S.moreleti* and the new species, and with *S.tamodienica* positioned as a basal lineage. Pairwise uncorrected COI p-distances among these three species also supported the species validity and ranged from 4.2% to 8.24%. Notably, *S.songkhramensis***sp. nov.** and *S.moreleti* exhibit similarities in shell morphology; however, the new species can be differentiated by more robust pseudocardinal teeth. Both species are distinguishable from *S.tamodienica* by their approximately twice larger size, more inflated shells, and more prominent, roughened, irregular growth lines on the shell surface. Furthermore, based on the current data, these three species are recognized as endemic and are restricted to disjunct biogeographic areas in Indochina: *S.tamodienica* in the Songkhla Lake Basin in southern Thailand, *S.moreleti* in the Tonle Sap and Lower Mekong basins, and *S.songkhramensis***sp. nov.** in the Songkhram Basin and its nearby tributaries of the Middle Mekong Basin.

## ﻿Introduction

Mainland Southeast Asia, also known as Indochina, is recognized as a world biodiversity hotspot for freshwater mussels (Unionoida), hosting a significant number of species and high levels of endemism (Graf and Cummings 2021), and thus has been hypothesized to be one of the origins for freshwater mussel radiation (Bolotov et al. 2017a). Indochina is characterized by its complex hydrological systems, which include at least three major freshwater catchments: the Salween in the west, the Chao Phraya in the middle, and the Mekong in the east, alongside several tributary systems in the coastal areas (Abell et al. 2008). These catchments are critical for the distribution and diversity of freshwater mussels, as they provide a variety of ecological niches and facilitate the evolution of endemic species (Konopleva et al. 2019b; Bolotov et al. 2020, 2022).

Two families of freshwater mussels are reported from Indochina. The Margaritiferidae, which are represented by only two species, are distributed exclusively in the northern part of the region (Brandt 1974; Lopes‐Lima et al. 2018). In contrast, the Unionidae exhibit a vast distribution range that encompasses the entire region, contributing to a notable richness in biodiversity with 144 species from 40 genera being recognized and approximately 80% of these species being considered endemic to this region (i.e., MUSSELp; Graf and Cummings 2024). Several of them have been recently described based on advancements in integrative taxonomy, combining morphological revisions, molecular data, and biogeographical history (Bolotov et al. 2017b, 2020; Konopleva et al. 2019b, 2021, 2023; Pfeiffer et al. 2021; Jeratthitikul et al. 2022, 2024; Jeratthitikul and Sutcharit 2023).

Recently, Konopleva et al. (2023) conducted a comprehensive phylogenetic investigation of the freshwater mussels in the Malay Peninsula Eastern Slope (southern Thailand), an area that has received less study compared to others, i.e., Chao Phraya and Mekong basins (Jeratthitikul et al. 2019a, 2019b, 2022; Konopleva et al. 2021; Pfeiffer et al. 2021). This study reveals several new taxa, including a monotypic genus, *Songkhlanaia*Konopleva et al., 2023, that was described based on a single specimen of the type species from Klong Tamod of the Songkhla Lake Basin (Konopleva et al. 2023: fig. 2c–h). The genus is characterized by a shell that is rectangular, rather compressed, posterior slope possessing distinct prominent folds, one pseudocardinal tooth on each valve, and lateral teeth absent (Konopleva et al. 2023). Apart from these conchological characteristics, the multi-locus phylogenetic analysis also revealed the genus as a distinct phylogenetic lineage, which is distantly related to other genera of the tribe Pseudodontini (Konopleva et al. 2023).

*Songkhlanaia* is considered to be restricted to the Songkhla Lake Basin (Bolotov et al. 2023; Konopleva et al. 2023), the largest lake in Thailand, which serves as an important ecological and economic resource for the surrounding communities (Cookey et al. 2016). However, the investigation of additional samples from the Mekong Basin, based on a combination of morphological characters and molecular phylogenetic analysis, has revealed two additional lineages within the genus. One of these coincides with a previously recognized species, while the other cannot be attributed to any known taxon; therefore, it is described herein as a new species. The diagnosis of the genus is also revised here to encompass the variation in shell morphology of the newly added species.

## ﻿Material and methods

### ﻿Specimen sampling

The animal use protocol in this study was approved by the Faculty of Science, Mahidol University Animal Care and Use Committee under approval number MUSC65-013-606 and MUSC66-016-646. Freshwater mussel specimens were collected by hand and euthanized at the collection site using the two-step method outlined by the AVMA (2020). Live specimens were initially placed in a container with freshwater. Then 95% (v/v) ethanol was gradually added to the container, starting at a concentration of approximately 5% (v/v) until the foot and adductor muscles relaxed completely. The anesthetized specimens were moved to another container with 70% (v/v) ethanol for fixation. Small pieces of foot tissues were snipped, preserved in 95% (v/v) ethanol, and stored at -20 °C for subsequent DNA extraction. The remaining specimens were dissected into soft body parts and shells. The soft body parts were stored in 70% (v/v) ethanol and used in anatomical study. The shells were kept as dry specimens. All specimens, including the type series of the new taxon, were deposited into the Mahidol University Museum of Natural History, Department of Biology, Faculty of Science, Mahidol University, Bangkok, Thailand (**MUMNH**).

### ﻿Morphological analysis

Species identification was based on shell characteristics following descriptions in the taxonomic literature (i.e., Crosse and Fischer 1876; Deshayes and Jullien 1876; Morlet 1884; Simpson 1914; Haas 1920, 1924, 1969; Konopleva et al. 2023) or by comparing with photographs of type series available on the online database of the Muséum national ďHistoire naturelle, Paris (**MNHN**; https://science.mnhn.fr). Various shell morphological characteristics were examined, including the outline, size, thickness, surface sculpture, shape and position of the umbo, hinge teeth structure, and muscle attachment scars. Shell dimensions were measured using a digital vernier caliper (±0.01 mm) for shell length, height, and width. Anatomical features of the soft body parts were also observed under a stereomicroscope.

### ﻿Molecular analysis

Genomic DNA was extracted from foot tissues using the NucleoSpin Tissue Extraction Kit (Macherey-Nagel, Germany) and stored at -20 °C for subsequent analysis. Partial fragments of the mitochondrial cytochrome c oxidase subunit I (COI), mitochondrial large ribosomal subunit rRNA (16S rRNA), and nuclear 28S large ribosomal subunit rRNA (28S rRNA) genes were amplified using polymerase chain reaction (PCR) and employed as genetic markers for phylogenetic analyses and genetic distance calculations (COI only). PCR primers, cycling conditions, and DNA sequencing were conducted following protocols established in our previous studies (Jeratthitikul et al. 2024). All newly generated sequences were deposited in the GenBank nucleotide sequence database under accession numbers PQ231666–PQ231681, PQ764574, and PQ764575 for COI; PQ236701–PQ236716, PQ776233, and PQ776234 for 16S rRNA; and PQ236717–PQ236732, PQ764576, and PQ764577 for 28S rRNA.

### ﻿Phylogenetic analysis

Phylogenetic trees were estimated using a concatenated dataset of the three aforementioned gene fragments generated from 70 mussel specimens (Suppl. material 1). The ingroup included 18 newly sequenced *Songkhlanaia* specimens from this study and the holotype of *Songkhlanaiatamodienica*, the type species (Konopleva et al. 2023). The outgroup was selected from previously published sequences of phylogenetic studies in the Unionidae (Huang et al. 2013; Pfeiffer and Graf 2015; Zieritz et al. 2016, 2021b; Bolotov et al. 2017a, 2017b, 2020, 2023; Lopes-Lima et al. 2017; Froufe et al. 2020; Jeratthitikul et al. 2021b, 2022, 2024; Konopleva et al. 2021, 2023; Jeratthitikul and Sutcharit 2023). These included single representative specimens of all available Pseudodontini taxa from GenBank (in total 44 species/subspecies), with representative species from other more distantly related unionid tribes (Schepmaniini, Gonideini, and Lamprotulini).

Separate multiple alignments for each gene were performed by the MUSCLE algorithm using MEGA11 v. 11.0.13 (Tamura et al. 2021), and later all three gene alignments were joined into one concatenated data matrix. The final concatenated data set was partitioned into five partitions (3 codons of COI + 16S rRNA + 28S rRNA). The optimal nucleotide substitution model for each partition was identified using PartitionFinder2 v. 2.3.4 (Lanfear et al. 2017) under the corrected Akaike Information Criterion (AICc). The program suggested GTR+I+G as the best nucleotide substitution model for the first codon of COI, 16S rRNA, and 28S rRNA partitions; F81+I for the second codon of COI partition; and GTR+G for the third codon of COI partition. These nucleotide substitution models were used in the subsequent phylogenetic analyses.

Phylogenetic analyses were conducted using Maximum Likelihood (ML) and Bayesian Inference (BI) methods on the online CIPRES Science Gateway (Miller et al. 2010). ML analysis was performed using IQ-TREE v. 2.2.2.7 (Minh et al. 2020) with 10,000 ultrafast bootstrap replicates to assess node support (Hoang et al. 2018). Bayesian Inference was conducted using MrBayes v. 3.2.7 (Ronquist et al. 2012) with four Markov Chain Monte Carlo (MCMC) chains run simultaneously for 10,000,000 generations. Tree samples were taken every 1,000 generations. The initial 25% of samples were discarded as burn-in. The average effective sample size (ESS) from the MCMC analysis was > 200 for all parameters. The resulting phylogenetic trees were visualized and edited using FigTree v. 1.4.4 (http://tree.bio.ed.ac.uk/software/figtree/). Nodes with ultrafast bootstrap support values (BS) ≥ 95% and Bayesian posterior probabilities (BPP) ≥ 0.95 were considered well-supported (San Mauro and Agorreta 2010; Hoang et al. 2018).

### ﻿Genetic distance analysis

Intraspecific and interspecific genetic distances were assessed using uncorrected p-distances calculated in MEGA11 v. 11.0.13 (Tamura et al. 2021) based on the mitochondrial COI gene dataset. The results are expressed as a percentage of the mean with standard deviation.

## ﻿Results

### ﻿Phylogenetic analysis and genetic distances

**Figure 1.**
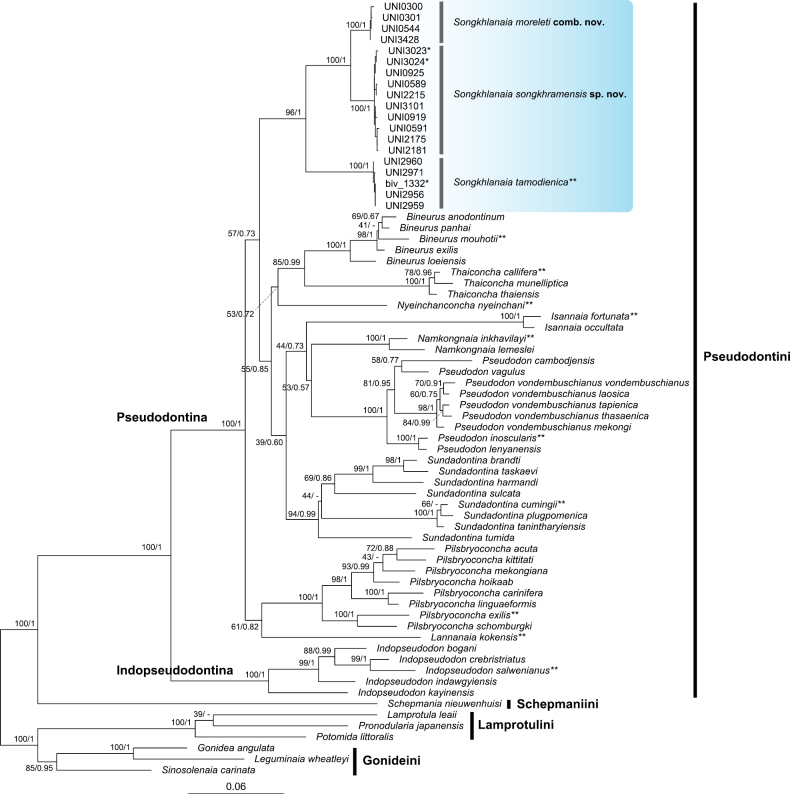
Maximum likelihood (ML) phylogenetic tree of freshwater mussels within the subfamily Gonideinae based on a combined DNA sequence dataset of COI, 16S rRNA, and 28S rRNA genes (1,959 bp). Branch support values are indicated on nodes as bootstrap percentages from ML analysis and Bayesian posterior probabilities from BI analysis, and shown as ML/BI. The scale bar represents the estimated evolutionary distance between taxa. The clade of the genus *Songkhlanaia* is highlighted in blue, and type specimens are indicated by an asterisk (*). Type species of Pseudodontini genera are marked with two asterisks (**).

Sequencing the target gene fragments from 19 specimens of *Songkhlanaia* produced 660 bp of COI, 481 bp of 16S rRNA, and 763–764 bp of 28S rRNA. After concatenated alignment of these three genes with outgroups, the final 1,959-bp matrix was generated and used for phylogenetic tree reconstruction. The ML and BI trees exhibited almost identical topologies; therefore, only the ML tree is depicted in Fig. 1. *Songkhlanaia* specimens form a single clade within the tribe Pseudodontini with significant supports for this relationship from both analyses (BS = 96%, BPP = 1). The genus is further divided into three strongly supported species-level clades (BS = 100%, BPP = 1), consisting of a clade of *S.tamodienica*, the type species of the genus, and two other novel clades recognized in this study. One clade, consisting of specimens collected from the Tonle Sap Basin, conchologically matches well with the previously recognized taxon, *Pseudodonmoreleti* Crosse & Fischer, 1876 (currently recognized as *Sundadontinamoreleti* by Bolotov et al. 2023). We thus recognize this clade as *Songkhlanaiamoreleti* comb. nov. Another clade consists of specimens collected from the Songkhram Basin and tributaries of the Mekong River in northeastern Thailand. This clade is conchologically similar to *S.moreleti* but possesses several diagnostic characteristics sufficient to separate them as distinct species (Table 1). It is thus described herein as *Songkhlanaiasongkhramensis* sp. nov.

**Table 1.** Comparison of conchological characteristics among *Songkhlanaia* species.

Conchological feature	*S.tamodienica* (*n* = 7)	*S.moreleti* (*n* = 3)	*S.songkhramensis* sp. nov. (*n* = 4; types)
Shell length (mm)	50.8–67.6	95.4–121.7	104.7–120.5
Shell height (mm)	32.9–43.7	62.0–78.7	73.8–78.5
Shell width (mm)	16.5–28.1	39.3–48.2	46.2–55.3
Shell shape	rectangular	subrhomboidal to ovate	subrhomboidal to ovate
Shell inflatedness	rather compressed	rather inflated	rather inflated
Shell thickness	not thick	moderate thick	thick
Shell color (adult specimens)	rusty brown to dark brown	dark brown to black	dark brown to black
Shell surface	fine growth lines, moderately roughened on the posterior slope	with irregular growth lines, roughened on the posterior slope and border of the shell	with irregular growth lines, roughened on the posterior slope and border of the shell
Folds on posterior slope	two fine folds	one or two faint folds	one or two faint folds
Umbo shape	tiny, slightly elevated	rounded, moderately elevated	rounded, wide, moderately elevated
Dorsal margin	straight, anterior low, posterior end high	slightly curved, anterior low, posterior end high	curved to slightly curved, anterior low, posterior end high
Ventral margin	slightly curved to almost straight	slightly curved to straight	slightly curved to straight
Right valve pseudocardinal tooth	tubercular to hill-like	tubercular or knob-like	triangular or high tubercular
Left valve pseudocardinal tooth	hill-like or lingula-shaped subcompressed	hill-like or triangulate, subcompressed	well-developed, rectangular, rather broad and high
V-shaped furrow on posterior end of the hinge structure	weak, not prominent	wide	wide
Anterior adductor muscle scar	shallow, somewhat droplet-like, contiguous with anterior protractor muscle scar	impressed, ovate, separated from anterior protractor muscle scar	impressed, somewhat drop-like, separated from anterior protractor muscle scar
Posterior adductor muscle scar	somewhat rounded, shallow to very shallow	drop-like to ovate, shallow	drop-like to ovate, shallow
Umbo cavity	shallow	moderate deep	deep, wide

The phylogenetic relationship between *S.moreleti* and the new species is significantly supported as sister taxa (BS = 100%, BPP = 1). Meanwhile, *S.tamodienica* was placed at the basal position within *Songkhlanaia*. Phylogenetic analyses further confirm *Songkhlanaia* as a member of the subtribe Pseudodontina with strong support (BS = 100%, BPP = 1). However, the phylogenetic position of this genus in relation to other genera is not significantly supported.

Interspecific divergence among *Songkhlanaia* species ranged from 4.17 to 8.26% uncorrected p-distance of the COI gene (Table 2), while intraspecific divergences were low, ranging from 0 to 0.34%.

**Table 2.** Mean genetic distances (uncorrected p-distance: %±SD) based on 660-bp COI fragment sequences among *Songkhlanaia* species (below diagonal), and within each species (in bold).

Taxon	1.	2.	3.
1. *S.tamodienica*	**0**		
2. *S.moreleti*	8.26 ± 0.07	**0.23 ± 0.02**	
3. *S.songkhramensis* sp. nov.	8.04 ± 0.18	4.17 ± 0.23	**0.34 ± 0.02**

### ﻿Taxonomic account

**Family Unionidae Rafinesque, 1820**

**Subfamily Gonideinae Ortmann, 1916**

**Tribe Pseudodontini Frierson, 1927**

**Subtribe Pseudodontina Frierson, 1927**

#### ﻿Genus Songkhlanaia

Taxon classificationAnimaliaUnionoidaUnionidae

Konopleva, Lheknim, Sriwoon, Kondakov, Vikhrev & Bolotov, 2023

5FDEEB17-1FA0-5F25-A9FA-0D099E66D316

Songkhlanaia Konopleva et al., 2023: 13, 14. Bolotov et al. 2023: 12.

##### Type species.

*Songkhlanaiatamodienica* Konopleva, Lheknim, Sriwoon, Kondakov, Vikhrev & Bolotov in Konopleva et al. 2023 (by original designation).

##### Species included.

*Songkhlanaia* currently consists of three species: *S.tamodienica* (type species), *S.moreleti* comb. nov., and *S.songkhramensis* sp. nov.

##### Diagnosis.

Shell medium to large, rectangular or subrhomboidal, rather compressed to inflated. Anteriorly constricted, dorsal margin straight or slightly curved, slightly elevated posteriorly. One or two folds on posterior slope. Shell surface with fine or irregular growth lines, roughened on posterior slope or border of shell. One pseudocardinal tooth on each valve; lateral teeth obsolete.

##### Distribution.

Endemic to Indochina, including Songkhla Lake Basin, Tonle Sap Basin, and Middle to Lower Mekong basins.

##### Comments.

*Songkhlanaia* is represented in multi-locus phylogenetic analyses as a distinct clade among the Pseudodontini genera (Fig. 1). Morphologically, its rectangular or overall subrhomboidal shape outline also makes *Songkhlanaia* easily distinguishable from other genera that have somewhat narrow and elongate shells: *Bineurus* Simpson, 1900, *Isannaia*Jeratthitikul et al., 2024, *Namkongnaia* Jeratthitikul et al., 2021, and *Pilsbryoconcha* Simpson, 1900 (Jeratthitikul et al. 2021b, 2024; Konopleva et al. 2021; Bolotov et al. 2023). Although members of *Songkhlanaia* possess rather short and high shell outlines which resemble those of the genera *Indopseudodon* Prashad, 1922, *Lannanaia*Jeratthitikul et al., 2024, *Nyeinchanconcha*Bolotov et al., 2020, *Pseudodon* Gould, 1844, *Sundadontina*Bolotov et al., 2020, and *Thaiconcha*Bolotov et al., 2020, the unique roughened and irregular growth lines on the outer shell surface (which are fine or less developed growth lines in *S.tamodienica*) make it easily distinguishable from these genera (Bolotov et al. 2020, 2023; Konopleva et al. 2021; Jeratthitikul et al. 2024).

Members of *Songkhlanaia* are likely the largest freshwater mussels in the tribe Pseudodontini recorded to date. The largest specimen examined herein is *S.moreleti* (MUMNH-UNI0301; shell length 121.7 mm) from Srakeo, Thailand. The syntype has a shell length of 123 mm (Crosse and Fischer 1876) and a specimen from Cambodia examined by Simpson (1914) reached 124 mm.

#### ﻿ Songkhlanaia tamodienica

Taxon classificationAnimaliaUnionoidaUnionidae

Konopleva, Lheknim, Sriwoon, Kondakov, Vikhrev & Bolotov, 2023

CFCEEF67-CC9F-5CE4-A8B9-B106AAACC3E3

**Figure 2.**
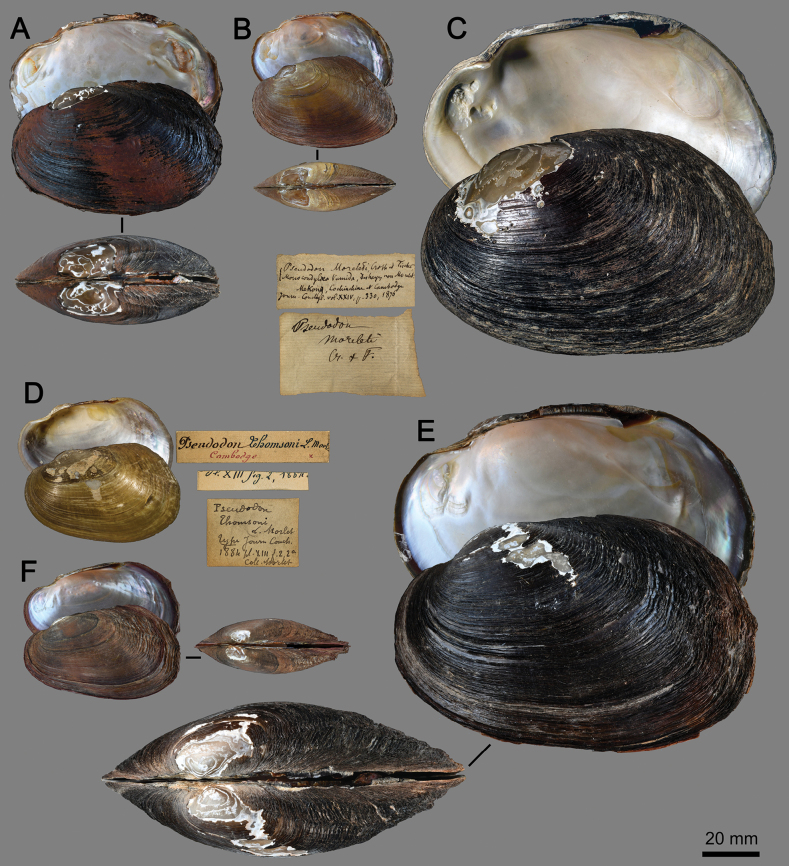
Shells of *Songkhlanaia* species **A, B** topotype of *S.tamodienica* from Tamot Stream, Songkhla Lake Basin, Thailand **A** adult specimen MUMNH-UNI2956 and **B** young specimen MUMNH-UNI2971 **C–F***S.moreleti***C** syntype MNHN-IM-2000–34623 from Mekong Basin, Cambodia, with original labels **D** syntype of *Pseudodonthomsoni* Morlet, 1884, MNHN-IM-2000-1800 from Mekong Basin, Cambodia, with original labels **E** adult specimen MUMNH-UNI0301, and **F** young specimen MUMNH-UNI3428 from Phrom Hot Stream, Tonle Sap Basin, Thailand. Photographs **C, D** M. Caballer (2019, MNHN Project: RECOLNAT No. ANR-11-INBS-0004).

Figs 2A, B , 5A , Table 1

Songkhlanaia tamodienica Konopleva et al., 2023: 14, fig. 2c–h. Type Locality: “Southern Thailand: Klong Plug Pom, middle reach of Klong Tamod, Songkhla Lake Basin, Ban Kok Sai, Tambon Mae Kree, Tamod District, Phatthalung Province, 7.3324°N, 100.0917°E”. Bolotov et al. 2023: 12.

##### Material examined.

Thailand – **Phatthalung Province** • 7 shells; Tamot District, Mae Khari Subdistrict, Songkhla Lake Basin, Tamot Stream; 7.3302°N, 100.0873°E; 17 May 2023; E. Jeratthitikul leg.; MUMNH-UNI2956 to 2960, UNI2971 to 2972.

##### Diagnosis.

Shell medium, rectangular, thin, rather compressed. Anteriorly constricted, dorsal margin straight, slightly elevated posteriorly. Posterior slope with two prominent folds. Umbo tiny, slightly elevated. Shell surface with fine irregular growth lines, roughened on posterior slope. Right valve with one smooth tubercular or triangular pseudocardinal tooth, left valve with somewhat lingula-shaped tooth. V-shaped furrow on posterior end of hinge structure weak, not prominent. Anterior adductor muscle scar shallow, somewhat drop-like, contiguous with anterior protractor muscle scar. Umbo cavity shallow.

##### Differential diagnosis.

This species can be distinguished from the other two congeners by its much smaller (about half size) and thin shell, rectangular shape, and rather compressed lateral profile. It can also be distinguished by 39 fixed nucleotide substitutions in the COI gene fragment (Table 3).

**Table 3.** Fixed nucleotide differences of COI sequences among *Songkhlanaia* species useful for species diagnosis. Nucleotide position based on the sequence alignment in this study.

Taxon	Fixed nucleotide differences
1. *S.tamodienica*	39G, 48A, 57A, 72A, 90C, 93G, 97T, 99G, 112C, 123G, 126C, 132A, 147A, 159C, 174G, 204C, 243A, 244T, 246G, 249C, 267G, 279C, 288A, 289T, 312A, 318C, 345T, 348A, 366C, 408T, 414G, 453A, 462G, 480A, 483A, 498T, 510A, 558A, 654T
2. *S.moreleti*	63A, 69A, 84A, 195C, 225G, 264A, 342C, 345A, 429C, 486C, 555A, 561G, 618T, 627T
3. *S.songkhramensis* sp. nov.	12C, 42C, 214G, 282A, 345G, 531T, 546T, 559C, 580C, 597C, 603C, 657A

##### Distribution.

**Figure 3.**
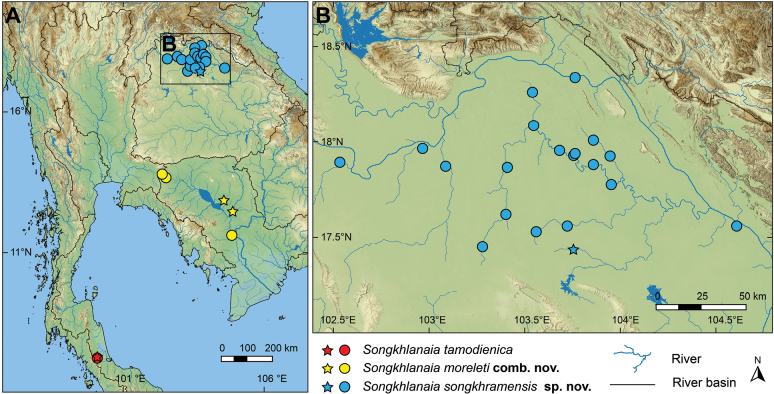
**A, B** map showing the geographical distribution of all known *Songkhlanaia* species (circles) and their type locality (stars). Map was generated using QGIS v. 3.36.0 with the outline of major river basins from the Freshwater Ecoregions of the World (Abell et al. 2008), river and lake topology from the HydroSHEDS database (https://www.hydrosheds.org), and the map raster data from the NASA EARTHDATA (https://www.earthdata.nasa.gov/).

So far, known only from the type locality in Songkhla Lake Basin, southern Thailand (Fig. 3).

##### Comments.

*Songkhlanaiatamodienica* was described based on a single specimen. The holotype has a relatively small (shell length 44.2 mm), thin, lighter colored shell with shallow adductor muscle scar, and slightly elevated posterior wing (Konopleva et al. 2023: fig. 2c–h). This specimen appeared to be small and young individual. Recently, we revisited the type locality and collected seven more specimens; two of these shells are larger in size and probably from fully grown specimens. They exhibit a thickened and inflated shell, rounded posterior end, dark periostracum, deep adductor muscle scar, and less pronounced posterior wing (Fig. 2A). The largest specimen measured is 67.3 mm in shell length. In addition, the outer shell surface is sculpted by irregular growth lines, which are pronounced on the posterior slope and border of the shell (Fig. 2A). This feature is present in younger specimens but is less prominent (Fig. 2B).

#### ﻿ Songkhlanaia moreleti

Taxon classificationAnimaliaUnionoidaUnionidae

(Crosse & Fischer, 1876) comb. nov.

9BDBEDA9-8C97-5EA7-9368-1649DEF9E8B2

Figs 2C–F , 5B , Table 1

Monocondylea [sic] *tumida* Deshayes & Jullien, 1876: 117–120, pl. 5, figs 1–3 [in part; non Monocondylustumidus Morelet, 1866].
Pseudodon moreleti Crosse & Fischer, 1876: 330. Type Locality: “les marécages qui avoisinent les rives du Mékong; dans un lac, à Préai-Bac, arroyo de Peam-Chelang; ďeau de la province de Compong-Soai” [= Peam Chileang and Kampong Svay, Cambodia]. Fischer 1891: 221. Fischer and Dautzenberg 1904: 436. Dautzenberg and Fischer 1905: 452, 453.
Pseudodon (Pseudodon) moreleti —Simpson 1900: 838. Simpson 1914: 1094, 1095. Haas 1924: 137, 138. Haas 1969: 130.
Sundadontina moreleti —Bolotov et al. 2020: 10, fig. 4e. Graf and Cummings 2021: 22. Bolotov et al. 2023: 13.
Pseudodon thomsoni Morlet, 1884: 401, 402, pl. 13, figs 2, 2a. Type Locality: “Cambodge” [= Cambodia]. Morlet 1889: 166. Fischer 1891: 221. Fischer-Piette 1950: 154. Zieritz et al. 2018: supplementary material 1. Bolotov et al. 2023: 11. Syn. nov.
Margaritana thomsoni —Paetel 1890: 174.
Pseudodon (Pseudodon) thomsoni —Simpson 1900: 838. Simpson 1914: 1092, 1093.
Pseudodon (Bineurus) thomsoni —Haas 1920: 336–337. Haas 1924: 144. Graf and Cummings 2007: 311.
?Pseudodonthomsoni—Konopleva et al. 2021: 11, fig. 3h.

##### Type material.

***Syntypes***MNHN-IM-2000–34623 (2 shells). ***Syntype***MNHN-IM-2000-1800 (1 shell) of *Pseudodonthomsoni* Morlet, 1884, (inadvertently stated as holotype by Fischer-Piette, 1950).

##### Other material examined.

Thailand – **Sa Kaeo Province** • 1 shell; Aranyaprathet District, Aranyaprathet Subdistrict, Tonle Sap Basin, Phrom Hot Stream; 13.6694°N, 102.5210°E; 31 Jan. 2015; E. Jeratthitikul leg.; MUMNH-UNI0544 • 1 shell; Aranyaprathet District, Aranyaprathet Subdistrict, Tonle Sap Basin, Phrom Hot Stream; 13.6718°N, 102.5166°E; MUMNH-UNI3428 • 2 shells; Watthana Nakhon District, Phak Kha Subdistrict, Tonle Sap Basin, Phrom Hot Stream; 13.7490°N, 102.4271°E; 5 May 2015; P. Prasankok leg.; MUMNH-UNI0300 to 0301.

##### Diagnosis.

Shell large, subrhomboidal to ovate, moderately thickened, rather inflated. Anteriorly constricted, dorsal slightly curved, slightly elevated posteriorly. Posterior slope with one or two faint folds. Umbo rounded, moderately elevated. Shell surface with irregular growth lines, roughened on posterior slope and border of shell. Right valve with one smooth tubercular pseudocardinal tooth, left valve with hill-like or triangulate pseudocardinal tooth, subcompressed. V-shaped furrow on posterior end of hinge structure prominent, wide. Anterior adductor muscle scar impressed, ovate, separated from anterior protractor muscle scar. Umbo cavity moderately deep.

##### Differential diagnosis.

This species is much larger and more inflated than the type species. Irregular growth lines on the shell surface are rougher, especially on the posterior slope and along the shell border. This species is also distinct from congenerics due to 14 fixed nucleotide substitutions in the COI gene fragment (Table 2).

##### Distribution.

Tonle Sap Basin in Thailand and Cambodia (Crosse and Fischer 1876; Deshayes and Jullien 1876; this study), and the Lower Mekong Basin in Cambodia (Morlet 1884) (Fig. 3).

##### Comments.

Originally, *Songkhlanaiamoreleti* was described based on a partially misidentified specimen from Cambodia as *Monocondylustumidus* Morelet, 1866 by Deshayes and Jullien (1876). Crosse and Fischer (1876) reexamined the specimens and provided it with a new name, *Pseudodonmoreleti*. This nominal species had been recognized as valid by subsequent studies for more than a hundred years (e.g., Fischer 1891; Fischer and Dautzenberg 1904; Dautzenberg and Fischer 1905; Simpson 1900, 1914; Haas 1924, 1969). Later, Brandt (1974: 271) and Graf and Cummings (2007: 311) listed this taxon as a junior synonym of either species or subspecies of ‘*tumidus* Morelet, 1866’. Recently, Bolotov et al. (2020) raised this species as valid and placed it in their new genus, *Sundadontina*. However, this resurrection and generic placement seemed provisionally based on conchological characters alone. Fortunately, specimens collected from Sa Kaeo Province in Thailand (Fig. 2E), the location of the headwaters of the Tonle Sap Basin and the type locality for this species, have been found to match well with the syntypes (Fig. 2C). Furthermore, these specimens cluster within the phylogenetic position of the *Songkhlanaia* (Fig. 1). Therefore, we propose transferring this species to the more appropriate genus *Songkhlanaia*.

*Pseudodonthomsoni* Morlet, 1884 was described based on specimens collected from Cambodia by Auguste Jean-Marie Pavie. Later, Morlet (1889: 166) detailed and specified the type locality as "Etang de Pnom-Penh (Cambodge)" [= pond in Phnom Penh, Cambodia]. It was recognized as a distinct species for more than a century, until it was recently treated as a junior synonym of *Thaiconchacallifera* (von Martens, 1860) by some authors (Bolotov et al. 2020: 10; Graf and Cummings 2021: 22). The following year, it was resurrected as a valid species by Konopleva et al. (2021) and again by Bolotov et al. (2023). However, the syntype of *Pseudodonthomsoni* Morlet, 1884 is relatively small (Fig. 2D; shell length 53 mm), and its shell characteristics generally resemble those of young specimens of *S.moreleti* (Fig. 2F), such as the long obovate shell that is constricted anteriorly, moderately elevated umbo, and a bean-shaped anterior protractor scar. Furthermore, the type locality in ‘Phnom Penh, Cambodia’ is in the lower Mekong Basin (Fig. 3), the same basin as the type locality of *S.moreleti*. Based on this conchological and biogeographic evidence, we thus synonymise this species with *S.moreleti*.

The molecular data examined in this study included individuals of *S.moreleti* solely collected from the headwater areas of the Tonle Sap Basin in Thailand. In fact, previous freshwater mollusk surveys of areas surrounding the Tonle Sap Lake in Cambodia by Ng et al. (2020) did not recover any specimens identified as *S.moreleti* from over 40 sampling localities. This possibly suggests a low abundance or local disappearance from the area. Further intensive surveys throughout the basin, including the headwater area and its tributaries, may encounter more specimens, which would be beneficial for assessing the genetic viability and conservation status of this species.

#### ﻿ Songkhlanaia songkhramensis sp. nov.

Taxon classificationAnimaliaUnionoidaUnionidae

F504AFD9-0E32-58EF-A2C7-C5A745CCB2C8

https://zoobank.org/B44C6882-92FD-460F-B904-CAD25A2222D6

**Figure 4.**
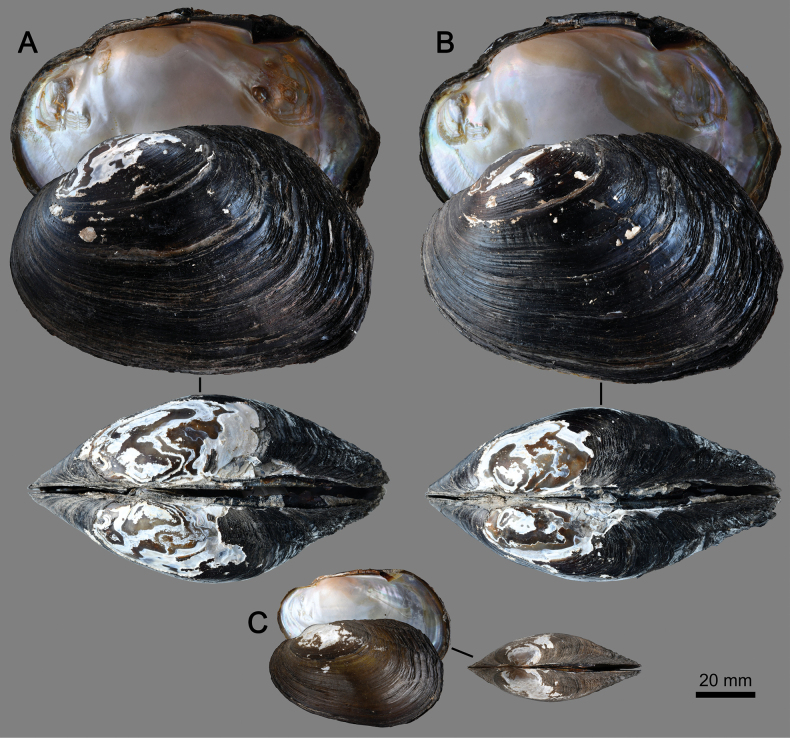
Shells of *Songkhlanaiasongkhramensis* sp. nov. **A** holotype MUMNH-UNI3024 and **B** paratype MUMNH-UNI3023 from Prahang River, Songkhram Basin, Thailand **C** specimen MUMNH-UNI0925 from Songkhram River, Songkhram Basin, Thailand.

**Figure 5.**
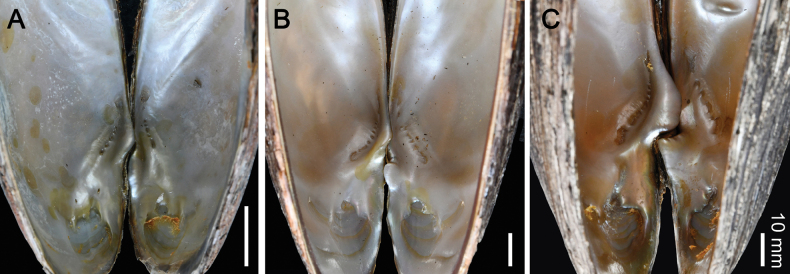
Pseudocardinal teeth, left valve on the left-hand side, and right valve on the right-hand side **A***S.tamodienica*, topotype MUMNH-UNI2956 from Tamot Stream, Songkhla Lake Basin, Thailand **B***S.moreleti*, specimen MUMNH-UNI0301 from Phrom Hot Stream, Tonle Sap Basin, Thailand **C***S.songkhramensis* sp. nov., holotype MUMNH-UNI3024 from Prahang River, Songkhram Basin, Thailand. Scale bars: 10 mm.

Figs 4 , 5C Tables 1 , 4

##### Type material.

***Holotype*** Thailand – **Sakon Nakhon Province** • Phang Khon District, Hai Yong Subdistrict, Songkhram Basin, Prahang River; 17.4376°N, 103.7569°E; 7 Apr. 2023; E. Jeratthitikul and W. Siriwut leg.; MUMNH-UNI3024 (shell length 120.5 mm, shell height 78.5 mm, shell width 55.3 mm). ***Paratype*** • 3 shells; same collection data as for holotype; MUMNH-UNI3022, 3023, 3025.

##### Other material.

Thailand – **Nong Khai Province** • 5 shells; Si Chiang Mai District, Nong Pla Pak Subdistrict, Mekong Basin, Nam Mong River; 17.8914°N, 102.5341°E; 7 Apr. 2015; E. Jeratthitikul, K. Wisittikoson, and P. Prasankok leg.; MUMNH-UNI2174 to 2178 • 2 shells; Phon Phisai District, Thung Luang Subdistrict, Mekong Basin, Nam Suai Stream; 17.9640°N, 102.9659°E; 8 Apr. 2015; E. Jeratthitikul, K. Wisittikoson, and P. Prasankok leg.; MUMNH-UNI2214, 2215. **Udon Thani Province** • 2 shells; Ban Dung District, Ban Dung Subdistrict, Songkhram Basin, Songkhram River; 17.8666°N, 103.4034°E; 5 Apr. 2023; E. Jeratthitikul and W. Siriwut leg.; MUMNH-UNI2978, 2979 • 2 shells; Ban Dung District, Ban Muang Subdistrict, Songkhram Basin, Songkhram River; 17.7293°N, 103.4101°E; 5 Apr. 2023; E. Jeratthitikul and W. Siriwut leg.; MUMNH-UNI2981, 2982 • 7 shells; Thung Fon District, Thung Fon Subdistrict, Songkhram Basin, Songkhram River; 17.4521°N, 103.2808°E; 8 Apr. 2015; E. Jeratthitikul, K. Wisittikoson, and P. Prasankok leg.; MUMNH-UNI919, 0925 to 0927, 3003 to 3005 • 3 shells; Sang Khom District, Chiang Da Subdistrict, Mekong Basin, Huai Luang River; 17.8730°N, 103.0875°E; 8 Apr. 2015; E. Jeratthitikul, K. Wisittikoson, and P. Prasankok leg.; MUMNH-UNI2179 to 2181. **Bueng Kan Province** • 6 shells; Mueang District, Khok Kong Subdistrict, Mekong Basin, Huay Kam Paeng. 18.3381°N, 103.7625°E; 5 Apr. 2015; E. Jeratthitikul, K. Wisittikoson, and P. Prasankok leg.; MUMNH-UNI0586 to 0591 • 3 shells; Seka District, Nong Thum Subdistrict, Songkhram Basin, Songkhram River; 17.8822°N, 103.8609°E; 4 Apr. 2023; E. Jeratthitikul and W. Siriwut leg.; MUMNH-UNI3051 to 3053 • 1 shell; Seka District, Pong Hai Subdistrict, Songkhram Basin, Nam Hee Stream, Unnamed Check Dam; 18.0117°N, 103.8585°E; 4 Apr. 2023; E. Jeratthitikul and W. Siriwut leg.; MUMNH-UNI3069 • 1 shell; Phon Charoen District, Wang Chomphu Subdistrict, Songkhram Basin, Songkhram River; 17.9557°N, 103.6802°E; 4 Apr. 2023; E. Jeratthitikul and W. Siriwut leg.; MUMNH-UNI3107 • 2 shells; Mueang District, Na Sawan Subdistrict, Songkhram Basin, Huay Pak Kong Stream, Ban Na Waeng Cheek Dam; 18.2603°N, 103.5425°E; 4 Apr. 2023; E. Jeratthitikul and W. Siriwut leg.; MUMNH-UNI3088, 3089 • 3 shells; Phon Charoen District, Nong Hua Chang Subdistrict, Songkhram Basin, Huay Pak Kong Stream, Unnamed Cheek Dam; 18.0863°N, 103.5462°E; 5 Apr. 2023; E. Jeratthitikul and W. Siriwut leg.; MUMNH-UNI3048 to 3050 • 9 shells; Seka District, Seka Subdistrict, Market (collected from Nam Hee Stream); 17.9265°N, 103.9455°E; 19 Jan 2023; K. Macharoenboon leg.; MUMNH-UNI2855 to 2863 • 9 shells; Seka District, Tha Sa-at Subdistrict, Songkhram Basin, Songkhram River; 17.9318°N, 103.7600°E; 5 Apr. 2015; E. Jeratthitikul, K. Wisittikoson, and P. Prasankok leg.; MUMNH-UNI0667, 0668. **Nakhon Phanom Province** • 2 shells; Tha Uthen District, Non Tan Subdistrict, Mekong Basin, Thuai River; 17.5621°N, 104.6096°E; 2 Apr. 2023; E. Jeratthitikul and W. Siriwut leg.; MUMNH-UNI3101, 3102. **Sakon Nakhon Province** • 2 shells; Sawang Daen Din District, Khok Si Subdistrict, Songkhram Basin, Songkhram River; 17.6205°N, 103.4020°E; 6 Apr. 2023; E. Jeratthitikul and W. Siriwut leg.; MUMNH-UNI3077, 3078 • 4 shells; Wanon Niwat District, Khon Sawan Subdistrict, Songkhram Basin, Yam Stream, Huai Kho Check Dam; 17.5637°N, 103.7203°E; 7 Apr. 2023; E. Jeratthitikul and W. Siriwut leg.; MUMNH-UNI3013 to 3016 • 7 shells; Charoen Sin District, Khok Sila Subdistrict, Songkhram Basin, Yam Stream; 17.5322°N, 103.5608°E; 3 May 2015; E. Jeratthitikul, K. Wisittikoson, and P. Prasankok leg.; MUMNH-UNI0344 to 0350 • 1 shell; Kham Ta Kla District, Kham Ta Kla Subdistrict, Songkhram Basin, Songkhram River; 17.9307°N, 103.7572°E; 18 Jan. 2023; K. Macharoenboon leg.; MUMNH-UNI2853 • 1 shell; Akat Amnuai District, Tha Kon Subdistrict, Songkhram Basin, Songkhram River; 17.7786°N, 103.9528°E; 9 Jan. 2023; local people leg.; MUMNH-UNI2198.

##### Diagnosis.

Shell large, subrhomboidal to ovate, thick, rather inflated. Anteriorly constricted, dorsal slightly curved, slightly elevated posteriorly. Posterior slope with one or two faint folds. Umbo rounded, wide, moderately elevated. Shell surface with irregular growth lines, roughened on posterior slope and border of shell. Right valve with one smooth triangular or high tubercular pseudocardinal tooth, left valve with well-developed, rectangular, rather broad and high pseudocardinal tooth. V-shaped furrow on posterior end of hinge structure prominent and wide. Anterior adductor muscle scar impressed, somewhat droplet-like, separated from anterior protractor muscle scar. Umbo cavity moderately deep and wide.

##### Differential diagnosis.

This new species can be distinguished from *S.moreleti* by having well-developed pseudocardinal teeth (Fig. 5), particularly the one on the left valve, which is characterized as a rectangular, rather broad, and high tooth (vs subcompressed, hill-like, or triangulate in *S.moreleti*); a wider V-shaped furrow at the posterior end of the hinge structure; and a deeper umbo cavity. The new species also possesses a set of unique fixed nucleotide substitutions in the COI gene fragment that make it genetically distinct from its congeners (Table 3). The new species is genetically closely related to *S.moreleti*, with a 4.17% uncorrected p-distance in the COI gene. They also form a sister clade in the phylogenetic analysis (Fig. 1).

##### Description.

Shell large-sized (shell length 104.7–120.5 mm, shell height 73.8–78.5 mm, shell width 46.2–55.3 mm; Table 4), thick, rather high (H/L ratio = 0.65–0.70), inequilateral, subrhomboidal to ovate shape, rather inflated. Anterior margin rounded; posterior margin oblique above, subtruncate below; ventral margin slightly curved to straight. Dorsal margin curved to slightly curved; anterior low, rather constricted, slightly elevated to posterior end; posterior end high, resembling posterior wing in young specimens (Fig. 4C). Umbo rounded, wide, moderately elevated, usually eroded. Posterior ridge wide and obtuse, not prominent; posterior slope with one or two faint folds running as curved line from umbo to posterior margin; lower one more prominent, ending at approximately middle of posterior margin, forming angulate point. Periostracum moderately thick, dark brown to black, eroded part white to coppery-brown. Shell surface with irregular growth lines, roughened on posterior slope and border of shell.

**Table 4.** Shell measurements and GenBank accession numbers for the type series of *Songkhlanaiasongkhramensis* sp. nov. Measurements in millimeters (mm).

Status of specimen	Specimen voucher	Shell dimensions (mm)			Genbank accession		
		length	height	width	COI	16S rRNA	28S rRNA
Holotype	MUMNH-UNI3024	120.5	78.5	55.3	PQ231674	PQ236709	PQ236725
Paratype	MUMNH-UNI3022	116.4	76.2	47.9	-	-	-
Paratype	MUMNH-UNI3023	109.8	76.3	53.5	PQ231673	PQ236708	PQ236724
Paratype	MUMNH-UNI3025	104.7	73.8	46.2	-	-	-

Inner side of shell: ligament long, narrow, dark brown in color. Pseudocadinal teeth one on each valve; smooth, triangular or high tubercular shape on right valve; well-developed, smooth, rectangular shape, rather broad and high on left valve; in shell coupling position, pseudocadinal tooth on right valve situated well anteriorly. Lateral teeth obsolete. Posterior end of hinge structure with wide V-shaped furrow. Anterior muscle scars impressed; anterior adductor muscle scar somewhat droplet-like, contiguous with anterior pedal retractor, but separated from anterior protractor muscle scars; pedal retractor muscle scar rounded, protractor muscle scar bean-shaped. Posterior adductor muscle scar large, drop-like to ovate, shallow. Pallial line well-marked and continuous. Umbo cavity deep, wide, with one row of 5–10 muscle scars. Nacre whitish blue to yellowish.

Siphon apertures with strip of dark pigmentation running along aperture edge. Exhalant aperture smooth, shorter than inhalant. Inhalant aperture with one row of conical papillae, varying in length, with more of the longer papillae near ventral edge. Small epithelial fold divides exhalant and inhalant aperture. Gills elongated and slightly ribbed; outer gills narrower than inner gills; anterior margin of inner gills slightly longer than outer gills. Labial palps elongate, somewhat pointed at tip. Glochidia unknown.

##### Etymology.

The species name *songkhramensis* refers to the Songkhram Basin, a sub-river basin of the Middle Mekong Basin in northeastern Thailand, in which this species is highly abundant. The type locality of the species is also situated in the Songkhram Basin.

##### Distribution.

The new species occurs in the Songkhram Basin and tributaries of the Mekong River in northeastern Thailand. It is a common freshwater mussel in the middle to upper part of Songkhram Basin (Fig. 3) and is usually found in high abundance.

##### Comments.

Among the mussel species commonly found sympatrically with *S.songkhramensis* sp. nov., *Thaiconchacallifera* is the most similar in overall shell features, and thus may confuse the identification. However, the new species can be easily distinguished from *T.callifera* by its thick shell (vs moderately thick), subrhomboidal to ovate shape (vs elliptical or rounded shape), higher shell (vs somewhat elongate), wider and more elevated umbo (vs narrow and slightly elevated), less shiny shell (vs somewhat shiny shell), shell surface sculptured with irregular growth lines, heavily roughened on the posterior slope (vs shell surface rather smooth, with fine growth lines, slightly roughened on the posterior slope), and rectangular and rather broad pseudocardinal teeth (vs tubercular pseudocardinal teeth) (Bolotov et al. 2020; Konopleva et al. 2021).

## ﻿Discussion

This study integrated molecular evidence, shell morphology, and biogeography into the identification of two additional species in the *Songkhlanaia*. One is a new combination of the previously recognized nominal taxon, *S.moreleti*, while the other is a new species from the Middle Mekong Basin, namely *S.songkhramensis* sp. nov. The discovery of the new species adds to the known diversity of the tribe Pseudodontini, making it the most speciose tribe of the Unionidae in Southeast Asia, with a total of 51 species across eleven genera, more than the 35 species in Contradentini and the 28 species in Gonideini (Graf and Cummings 2021, 2024; Bolotov et al. 2023; Jeratthitikul et al. 2024). Moreover, the discovery of this new species further emphasizes the remarkable diversity and endemism of the freshwater mussel fauna in the Mekong Basin, and particularly in the Songkhram Basin, the recently listed Ramsar site in Thailand. The Songkhram Basin houses a diverse assemblage of unionid mussels accounting for 12 species from 12 genera; nine of these species (75%) are considered as endemic to the basin (Jeratthitikul et al. 2019a, 2019b, 2021a, 2021b, 2024; Muanta et al. 2019; Konopleva et al. 2021; Pfeiffer et al. 2021; Bolotov et al. 2023). Unfortunately, the high levels of endemism in this area are threatened by anthropogenic impacts, including pollution and habitat destruction (Saluja and Piman 2022), which may result in significant habitat loss for the mussels, thereby threatening their survival (Lopes-Lima et al. 2018; Aldridge et al. 2023).

Members of the Pseudodontini share common characteristics of the obsolete lateral teeth and the single pseudocardinal tooth on each valve, which can be represented as either weakly or well-developed knob-like pseudocardinal teeth. Meanwhile, other conchological traits exhibit a broad range of variability, and species in the taxon range from having thin and ultra-elongate shells to rather thick and rounded shells (Bolotov et al. 2023). Species in each genus of the Pseudodontini also exhibit high levels of cryptic diversity, rendering it challenging to distinguish them based solely on morphological characteristics (Jeratthitikul et al. 2022; Bolotov et al. 2023). This challenge is particularly evident in the comparison between two sister species, *S.moreleti* and *S.songkhramensis* sp. nov. Although these species can be differentiated by their pseudocardinal teeth, they are very similar in overall shell features (Table 1). However, we found that fragments of the COI gene, the common DNA barcoding gene marker used in freshwater mussels (e.g., Pfeiffer et al. 2021; Jeratthitikul et al. 2022, 2024; Jeratthitikul and Sutcharit 2023), remains an effective tool for distinguishing between them. They are genetically different by 4.17% uncorrected p-distance of the COI gene (Table 2) and have several fixed nucleotide differences (Table 3). This genetic divergence is comparable to established thresholds for species delimitation in other Indochinese freshwater mussels ranging from 2.32 to 12.3% (Jeratthitikul et al. 2019a, 2019b, 2021a, 2021b, 2022, 2024; Konopleva et al. 2019a; Jeratthitikul and Sutcharit 2023). This study thus has once again highlighted the importance of utilizing an integrative approach of combining morphology and molecular data in species delimitation of freshwater mussels.

Multi-locus phylogenetic analysis (COI + 16S rRNA + 28S rRNA) in this study recovered members of *Songkhlanaia* as a well-supported clade (Fig. 1), confirming the identity of the genus among the Pseudodontini genera. Furthermore, the genus was placed in the subtribe Pseudodontina with significant support from both ML and BI analyses (BS = 100%, BPP = 1). However, the phylogenetic position of this genus in relation to other genera in the Pseudodontina remains unstable among studies that use similar genetic markers. The Bayesian time-calibrated phylogenetic tree in Bolotov et al. (2023) suggested a separation of *Pilsbryoconcha* from other genera in the Pseudodontina, including *Songkhlanaia*, although the relationships among the genera within this clade were uncertain. Jeratthitikul et al. (2024) revealed a supported sister relationship between *Songkhlanaia* and *Lannanaia*. In contrast, the phylogenetic results in Konopleva et al. (2023) and this study fail to recover a supported phylogenetic position for *Songkhlanaia*. This incongruent phylogenetic relationship suggests that using these three genetic markers may not be sufficient to recover strong support for the deep nodes within Pseudodontina. To enhance phylogenetic resolution, further studies should incorporate longer sequences (i.e., the whole 28S rRNA gene), add more genes such as ND1, histone 3, or 18s rRNA (Ortiz-Sepulveda et al. 2020; Zieritz et al. 2024), or utilize complete mitochondrial genomes (Froufe et al. 2020; Zieritz et al. 2021a), as well as employ more comprehensive phylogenomic datasets (Pfeiffer et al. 2019).

Alternatively, the unclear relationships among genera within the Pseudodontina might be attributed to rapid radiation within the group, where lineages may have undergone a series of speciation events in a relatively short period of time. This phenomenon could result in a complex evolutionary history that complicates the resolution of phylogenetic trees with certain features such as short internal branches and poorly supported nodes, as evidenced in other groups of animals (e.g., Grummer et al. 2018; Duan et al. 2023). The time estimation of rapid radiation events of the Pseudodontina genera has been suggested to occur during the Late Cretaceous to Eocene times (approximately 75–50 million years; Jeratthitikul et al. 2021b; Bolotov et al. 2023).

## Supplementary Material

XML Treatment for Songkhlanaia

XML Treatment for Songkhlanaia tamodienica

XML Treatment for Songkhlanaia moreleti

XML Treatment for Songkhlanaia songkhramensis

## ﻿Additional information

### Author ORCIDs

Ekgachai Jeratthitikul https://orcid.org/0000-0002-3477-9548

Chirasak Sutcharit https://orcid.org/0000-0001-7670-9540

Pongpun Prasankok https://orcid.org/0000-0001-5967-223X

### Data availability

All of the data that support the findings of this study are available in the main text or Supplementary Information.

## Supplementary materials

Supplementary material 1 List of voucher specimens with GenBank accession numbers used in phylogenetic analysis Data type docx https://binary.pensoft.net/file/1236498This dataset is made available under the Open Database License (http://opendatacommons.org/licenses/odbl/1.0/). The Open Database License (ODbL) is a license agreement intended to allow users to freely share, modify, and use this Dataset while maintaining this same freedom for others, provided that the original source and author(s) are credited.Ekgachai Jeratthitikul, Chirasak Sutcharit, Pongpun Prasankok
