# Supplementary material for: ﻿An integrative approach to a revision of the freshwater mussel genus Songkhlanaia (Bivalvia, Unionidae), with the description of a new species
Source: Zookeys. 2025 Jan 28;1224:187–209. doi: 10.3897/zookeys.1224.140549 (PMC11795186; doi:10.3897/zookeys.1224.140549)
Supplement: Supplementary material 1 — List of voucher specimens with GenBank accession numbers used in phylogenetic analysis [file zookeys-1224-187_article-140549__-s001.docx]

**Supplementary Table S1** List of taxa used in phylogenetic analyses with GenBank accession numbers.

| **Taxa** | **Voucher ID** | **Locality** | **GenBank accession** | | | **Reference** |
| --- | --- | --- | --- | --- | --- | --- |
|  |  |  | **COI** | **16S rRNA** | **28S rRNA** |  |
| **Tribe Pseudodontini Frierson, 1927** | | | | | | |
| **Subtribe Pseudodontina Frierson, 1927** | | | | | | |
| *Songkhlanaia tamodienica* Konopleva et al., 2023 | RMBH biv 1332 | Thailand: Mekong Basin, Klong Tamod Stream | OP729832 | OP735386 | OP735369 | [1] |
| *Songkhlanaia tamodienica* Konopleva et al., 2023 | MUMNH:UNI2956 | Thailand: Songkhla Lake Basin, Tamot Stream | PQ231666 | PQ236701 | PQ236717 | This study |
| *Songkhlanaia tamodienica* Konopleva et al., 2023 | MUMNH:UNI2959 | Thailand: Songkhla Lake Basin, Tamot Stream | PQ231667 | PQ236702 | PQ236718 | This study |
| *Songkhlanaia tamodienica* Konopleva et al., 2023 | MUMNH:UNI2960 | Thailand: Songkhla Lake Basin, Tamot Stream | PQ231668 | PQ236703 | PQ236719 | This study |
| *Songkhlanaia tamodienica* Konopleva et al., 2023 | MUMNH:UNI2971 | Thailand: Songkhla Lake Basin, Tamot Stream | PQ231669 | PQ236704 | PQ236720 | This study |
| *Songkhlanaia moreleti* (Crosse & P. Fischer, 1876) **comb. nov.** | MUMNH:UNI0300 | Thailand: Tonle Sap Basin, Phrom Hot Stream | PQ231670 | PQ236705 | PQ236721 | This study |
| *Songkhlanaia moreleti* (Crosse & P. Fischer, 1876) **comb. nov.** | MUMNH:UNI0301 | Thailand: Tonle Sap Basin, Phrom Hot Stream | PQ231671 | PQ236706 | PQ236722 | This study |
| *Songkhlanaia moreleti* (Crosse & P. Fischer, 1876) **comb. nov.** | MUMNH:UNI0544 | Thailand: Tonle Sap Basin, Phrom Hot Stream | PQ231672 | PQ236707 | PQ236723 | This study |
| *Songkhlanaia moreleti* (Crosse & P. Fischer, 1876) **comb. nov.** | MUMNH:UNI3428 | Thailand: Tonle Sap Basin, Phrom Hot Stream | PQ764575 | PQ776234 | PQ764577 | This study |
| *Songkhlanaia songkhramensis* **sp. nov.** | MUMNH:UNI3023 | Thailand: Songkhram Basin, Prahang Stream | PQ231673 | PQ236708 | PQ236724 | This study |
| *Songkhlanaia songkhramensis* **sp. nov.** | MUMNH:UNI3024 | Thailand: Songkhram Basin, Prahang Stream | PQ231674 | PQ236709 | PQ236725 | This study |
| *Songkhlanaia songkhramensis* **sp. nov.** | MUMNH:UNI3101 | Thailand: Mekong Basin, Thuai River | PQ231675 | PQ236710 | PQ236726 | This study |
| *Songkhlanaia songkhramensis* **sp. nov.** | MUMNH:UNI0919 | Thailand: Songkhram Basin, Songkhram River | PQ231676 | PQ236711 | PQ236727 | This study |
| *Songkhlanaia songkhramensis* **sp. nov.** | MUMNH:UNI0589 | Thailand: Mekong Basin, Huay Kam Paeng | PQ231677 | PQ236712 | PQ236728 | This study |
| *Songkhlanaia songkhramensis* **sp. nov.** | MUMNH:UNI0591 | Thailand: Mekong Basin, Huay Kam Paeng | PQ231678 | PQ236713 | PQ236729 | This study |
| *Songkhlanaia songkhramensis* **sp. nov.** | MUMNH:UNI2215 | Thailand: Mekong Basin, Nam Suai Stream | PQ231679 | PQ236714 | PQ236730 | This study |
| *Songkhlanaia songkhramensis* **sp. nov.** | MUMNH:UNI2175 | Thailand: Mekong Basin, Nam Mong River | PQ231680 | PQ236715 | PQ236731 | This study |
| *Songkhlanaia songkhramensis* **sp. nov.** | MUMNH:UNI2181 | Thailand: Mekong Basin, Huai Luang River | PQ231681 | PQ236716 | PQ236732 | This study |
| *Songkhlanaia songkhramensis* **sp. nov.** | MUMNH:UNI0925 | Thailand: Mekong Basin, Songkhram River | PQ764574 | PQ776233 | PQ764576 | This study |
| *Bineurus anodontinum* (Rochebrune, 1882) | UF 507391 (ICH-00445) | Cambodia: Mekong Basin, Tonle Sekong River | MW603639 | n/a | n/a | [2] |
| *Bineurus exilis* (Morelet, 1866) | RMBH biv0474_1 | Thailand: Mekong Basin, Mun River | MN275052 | MN307243 | MN307184 | [3] |
| *Bineurus loeiensis* Konopleva et al., 2021 | biv119_1 | Thailand: Mekong Basin, Loei River | KX865879 | KX865650 | KX865750 | [2] |
| *Bineurus mouhotii* (Lea, 1863) | RMBH biv0182_2 | Laos: Mekong Basin, Nam Long River | KX865876 | KX865647 | KX865747 | [4] |
| *Bineurus panhai* Jeratthitikul & Sucharit, 2023 | MUMNH-UNI2840 | Thailand: Bang Pakong Basin, Phra Sathueng Stream | OQ108585 | OQ110575 | OQ110565 | [5] |
| *Isannaia fortunata* Jeratthitikul, Sucharit & Prasankok, 2024 | MUMNH-UNI1950 | Thailand: Mekong Basin, Thuai River | OR987590 | OR987516 | OR987525 | [6] |
| *Isannaia occultata* Jeratthitikul, Sucharit & Prasankok, 2024 | MUMNH-UNI2895 | Thailand: Mekong Basin, Yang Stream | OR987594 | OR987520 | OR987529 | [6] |
| *Lannanaia kokensis* Jeratthitikul, Sucharit & Prasankok, 2024 | MUMNH-UNI0825 | Thailand: Mekong Basin, Kok River | OR987589 | OR987515 | OR987524 | [6] |
| *Namkongnaia inkhavilayi* Jeratthitikul et al., 2021 | MUMNH-UNI2831 | Laos: Mekong Basin, Xe Bangfai River | MZ822395 | MZ822895 | MZ822917 | [7] |
| *Namkongnaia lemeslei* (Morelet, 1875) | MUMNH-UNI2825 | Cambodia: Tole Sap Basin, Kampong Kdei River | MZ822399 | MZ822899 | MZ822921 | [7] |
| *Nyeinchanconcha nyeinchani* Bolotov et al., 2020 | UMMZ 304648 | Laos: Mekong River basin, Nam Phiat River | KP795025 | KP795050 | KP795008 | [8] |
| *Pilsbryoconcha acuta* Jeratthitikul & Prasankok, 2022 | MUMNH-UNI1510 | Thailand: Mekong Basin, Dom Yai River | OP589124 | OP595955 | OP595884 | [9] |
| *Pilsbryoconcha carinifera* (Conrad, 1837) | MUMNH-UNI2823 | Thailand: Tonle Sap Basin, Yang Stream | OP589099 | OP595930 | OP595859 | [9] |
| *Pilsbryoconcha exilis* (Lea, 1838) | MUMNH-UNI2481 | Indonesia: Java Basin, Bogor Botanical Gardens | MZ822408 | MZ822908 | MZ822930 | [7] |
| *Pilsbryoconcha hoikaab* Jeratthitikul & Prasankok, 2022 | MUMNH-UNI0305 | Thailand: Mekong Basin, Kam River | OP589107 | OP595938 | OP595867 | [9] |
| *Pilsbryoconcha kittitati* Jeratthitikul & Prasankok, 2022 | MUMNH-UNI0372 | Thailand: Mekong Basin, Udon Thani | OP589119 | OP595950 | OP595879 | [9] |
| *Pilsbryoconcha linguaeformis* (Morelet, 1875) | MUMNH-UNI2625 | Cambodia: Tole Sap Basin,Tole Sap Lake | MZ822413 | MZ822913 | MZ822935 | [7] |
| *Pilsbryoconcha mekongiana* Jeratthitikul & Prasankok, 2022 | MUMNH-UNI0843 | Thailand: Mekong Basin, Bueng Kan | OP589114 | OP595945 | OP595874 | [9] |
| *Pilsbryoconcha schomburgki* (Martens, 1860) | MUMNH-UNI0582 | Thailand: Mae Klong River | OP589086 | OP595917 | OP595846 | [9] |
| *Pseudodon cambodjensis* (Petit, 1865) | RMBH biv0811_1 | Laos: Mekong Basin, Nam Ngum River | OQ836225 | OQ832370 | OQ832334 | [10] |
| *Pseudodon inoscularis* (Gould, 1844) | RMBH biv1027_1 | Myanmar: Dawei (Tavoy) River basin, Yae Pone Stream | OQ836329 | OQ832401 | OQ832365 | [10] |
| *Pseudodon lenyanensis* Bolotov et al., 2020 | RMBH biv0628_2 | Myanmar: Lenya Basin | MN275055 | MN307246 | MN307187 | [3] |
| *Pseudodon vagulus* (Fischer, 1891) | UMMZ 304350 | Cambodia: Mekong River basin Pursat River, a tributary of Tonle Sap River, | KP795028 | KF011262 | KP795011 | [8] |
| *Pseudodon vondembuschianus vondembuschianus* (Lae, 1840) | BIV1822 | Malaysia | MK994774 | MK994774 | MZ684028 | [11, 12] |
| *Pseudodon vondembuschianus laosica* Bolotov et al., 2020 | UMMZ 304650 | Laos: Mekong basin, tributary of Vang Ngao River | KP795029 | KP795029 | n/a | [8] |
| *Pseudodon vondembuschianus mekongi* Bolotov et al., 2020 | RMBH biv0122 | Thailand: Mekong Basin, Phong River | KX865861 | KX865632 | KX865733 | [4] |
| *Pseudodon vondembuschianus tapienica* Konopleva et al., 2023 | RMBH biv1272_2 | Thailand: Klong Min River | OP729819 | OP735378 | OP735361 | [1] |
| *Pseudodon vondembuschianus thasaenica* Konopleva et al., 2023 | RMBH biv1321_1 | Thailand: Tha Taphao River Basin, Klong Thasae Stream | OP729822 | OP735379 | OP735364 | [1] |
| *Thaiconcha callifera* (Martens, 1860) | RMBH biv0120_3 | Thailand: Mekong Basin, Phong River | KX865865 | KX865636 | KX865737 | [4] |
| *Thaiconcha munelliptica* Konopleva et al., 2021 | biv462 | Thailand: Mekong Basin, Mun River | MN275063 | MN307252 | MN307193 | [3] |
| *Thaiconcha thaiensis* Konopleva et al., 2021 | UF 567706 (2014-0700) | Thailand: Mekong Basin, Kham Nong Bua River | MW603630 | n/a | MW647150 | [2] |
| *Sundadontina brandti* Bolotov et al., 2020 | RMBH biv0475_2 | Thailand: Mekong Basin, Mun River | MN275058 | MN307249 | MN307190 | [3] |
| *Sundadontina cumingii* (Lea, 1851) | X115 | Malaysia | KX051295 | n/a | n/a | [13] |
| *Sundadontina harmandi* (Crosse & Fischer, 1876) | RMBH biv0881_1 | Laos: Mekong River basin, Huay Tuay River | OQ836287 | OQ832387 | OQ832351 | [10] |
| *Sundadontina plugpomenica* Konopleva et al., 2023 | RMBH biv 1324/1 | Thailand: Klong Pa-Payom Stream | OP729828 | OP735383 | OP735367 | [1] |
| *Sundadontina sulcata* (Rochebrune, 1882) | RMBH biv0869_1 | Laos: Mekong Basin, Phaphou Stream | OQ836273 | OQ832381 | OQ832345 | [10] |
| *Sundadontina tanintharyiensis* Bolotov et al., 2020 | RMBH biv0643_4 | Myanmar: Lenya Basin, Chaung Nauk Pyan Stream | MN275057 | MN307248 | MN307189 | [3] |
| *Sundadontina taskaevi* Bolotov et al., 2020 | RMBH biv0475_1 | Thailand: Mekong River, Mun River | MN275061 | MN307251 | MN307192 | [3] |
| *Sundadontina tumida* (Morelet, 1866) | UMMZ 304349 | Cambodia: Mekong Basin | KP795027 | KF011261 | KP795010 | [8]; GenBank |
| **Subtribe Indopseudodontina Bolotov et al., 2023** | | | | | | |
| *Indopseudodon bogani* (Bolotov, Kondakov & Konopleva, 2017) | RMBH biv0241_4 | Myanmar: Sittaung Basin, Kanni River | MF352216 | MF352290 | MF352348 | [14] |
| *Indopseudodon crebristriatus* (Anthony, 1865) | RMBH biv0986_1 | Myanmar: Bago River | OQ836319 | OQ832397 | OQ832361 | [10] |
| *Indopseudodon indawgyiensis* Bolotov et al., 2023 | RMBH biv0110_10 | Myanmar: Ayeyarwady Basin, a tributary of Lake Indawgyi | KX865858 | KX865629 | KX865730 | [4] |
| *Indopseudodon kayinensis* (Bolotov et al., 2020) | RMBH biv0618_3 | Myanmar: Ataran Basin, Winyaw River | MN275045 | MN307240 | MN307181 | [3] |
| *Indopseudodon salwenianus* (Gould, 1844) | RMBH biv0674_1 | Myanmar: Salween Basin, Hlaingbwe Stream | MN275040 | MN307238 | MN307179 | [3] |
| **Tribe Gonideini Ortmann, 1916** | | | | | | |
| *Gonidea angulata* (Lea, 1838) | RMBH biv0294_1 | USA: Okanagan Lake | MN402615 | MN396726 | MN396722 | [3] |
| *Leguminaia wheatleyi* (Lea, 1862) | RMBH biv0177_7 | Turkey: Karasu River | MN402614 | MN396725 | MN396721 | [3] |
| *Sinosolenaia carinata* (Heude, 1877) | n/a | China | KX822669 | NC_023250 | KX822626 | [15, 16] |
| **Tribe Lamprotulini Modell, 1942** | | | | | | |
| *Lamprotula leaii* (Gray, 1833) | RMBH biv0200_1 | Vietnam | MN402616 | MN396727 | MN396723 | [3] |
| *Potomida littoralis* (Cuvier, 1798) | RMBH biv0177_10 | Turkey: Karasu River | MN402617 | MN396728 | MN396724 | [3] |
| *Pronodularia japanensis* (Lea, 1859) | NCSM 27183 | Japan | KX822659 | AB055625 | KX822615 | [15]; GenBank |
| **Tribe Schepmaniini Lopes-Lima, Pfeiffer & Zieritz, 2021** | | | | | | |
| *Schepmania nieuwenhuisi* (Schepman, 1898) | BOR MOL 14421_x475 | Malaysia: Borneo | MZ678755 | MZ684082 | MZ684035 | [12] |

**References**

1. Konopleva, E.S., et al., *Diversity and phylogenetics of freshwater mussels (Unionidae) from southern Thailand with the description of one new genus and five new species-group taxa.* Diversity, 2023. **15**: p. 10.

2. Konopleva, E.S., et al., *New freshwater mussels from two Southeast Asian genera Bineurus and Thaiconcha (Pseudodontini, Gonideinae, Unionidae).* Scientific Reports, 2021. **11**(1): p. 8244.

3. Bolotov, I.N., et al., *New freshwater mussel taxa discoveries clarify biogeographic division of Southeast Asia.* Scientific Reports, 2020. **10**(1): p. 6616.

4. Bolotov, I.N., et al., *Ancient river inference explains exceptional oriental freshwater mussel radiations.* Scientific Reports, 2017. **7**: p. 2135.

5. Jeratthitikul, E. and C. Sutcharit, *Multi-locus phylogeny reveals a new freshwater mussel in the genus Bineurus Simpson, 1900 (Unionidae: Pseudodontini) from Thailand.* Tropical Natural History, 2023. **Supplement 7**: p. 173–180.

6. Jeratthitikul, E., C. Sutcharit, and P. Prasankok, *Two new genera and three new species of exceptionally rare and endemic freshwater mussels (Bivalvia: Unionidae) from the Mekong Basin.* Zoosystematics and Evolution, 2024. **100**(4): p. 1333–1345.

7. Jeratthitikul, E., et al., *Molecular phylogeny reveals a new genus of freshwater mussels from the Mekong River Basin (Bivalvia: Unionidae).* European Journal of Taxonomy 2021. **775**: p. 119–142.

8. Pfeiffer, J.M. and D.L. Graf, *Evolution of bilaterally asymmetrical larvae in freshwater mussels (Bivalvia: Unionoida: Unionidae).* Zoological Journal of the Linnean Society, 2015. **175**(2): p. 307–318.

9. Jeratthitikul, E., et al., *Phylogeny and biogeography of Indochinese freshwater mussels in the genus Pilsbryoconcha Simpson, 1900 (Bivalvia: Unionidae) with descriptions of four new species.* Scientific Reports, 2022. **12**(1): p. 20458.

10. Bolotov, I.N., et al., *Integrative taxonomic reappraisal and evolutionary biogeography of the most diverse freshwater mussel clade from Southeast Asia (Pseudodontini).* Water, 2023. **15**: p. 3117.

11. Froufe, E., et al., *Mesozoic mitogenome rearrangements and freshwater mussel (Bivalvia: Unionoidea) macroevolution.* Heredity, 2020. **124**(1): p. 182–196.

12. Zieritz, A., et al., *A new genus and two new, rare freshwater mussel (Bivalvia: Unionidae) species endemic to Borneo are threatened by ongoing habitat destruction.* Aquatic Conservation: Marine and Freshwater Ecosystems, 2021. **31**(11): p. 3169–3183.

13. Zieritz, A., et al., *Factors driving changes in freshwater mussel (Bivalvia, Unionida) diversity and distribution in Peninsular Malaysia.* Science of The Total Environment, 2016. **571**: p. 1069–1078.

14. Bolotov, I.N., et al., *New taxa of freshwater mussels (Unionidae) from a species-rich but overlooked evolutionary hotspot in Southeast Asia.* Scientific Reports, 2017. **7**: p. 11573.

15. Lopes-Lima, M., et al., *Phylogeny of the most species-rich freshwater bivalve family (Bivalvia: Unionida: Unionidae): Defining modern subfamilies and tribes.* Molecular Phylogenetics and Evolution, 2017. **106**: p. 174–191.

16. Huang, X.-C., et al., *The complete maternally and paternally inherited mitochondrial genomes of the endangered freshwater mussel Solenaia carinatus (Bivalvia: Unionidae) and implications for Unionidae taxonomy.* PLOS ONE, 2013. **8**(12): p. e84352.
